# The comparison of effectiveness of acupressure on Spleen 6 and Hugo points on the severity of postpartum pain: A randomized clinical trial

**DOI:** 10.1002/hsr2.2265

**Published:** 2024-07-18

**Authors:** Saba Mohamadghasem‐Nejad Maleki, Sousan Heydarpour, Rojin Nikrai, Fateme Heydarpour

**Affiliations:** ^1^ Student Research Committee Kermanshah University of Medical Sciences Kermanshah Iran; ^2^ Department of Reproductive Health, School of Nursing and Midwifery Kermanshah University of Medical Sciences Kermanshah Iran; ^3^ Department of Physical Medicine and Rehabilitation and Research Center, Shohada‐e Tajrish Hospital, School of Medicine Shahid Beheshti University of Medical Sciences Tehran Iran; ^4^ Social Development & Health Promotion Research Center, Health Institute Kermanshah University of Medical Sciences Kermanshah Iran

**Keywords:** Hugo point acupressure, severity of postpartum pain, Spleen 6 acupressure

## Abstract

**Background and aims:**

Postpartum pain poses a significant challenge for new mothers. Various nonpharmacological methods are employed to manage postpartum pain. This study aimed to compare the effectiveness of acupressure on Spleen 6 and Hugo points on the severity of postpartum pain.

**Methods:**

In this parallel randomized trial study, 68 eligible primiparous women who had vaginal deliveries and experienced postpartum pain at Farabi Hospital in Malekan (a city in East Azarbaijan Province in Iran) were selected according to inclusion/exclusion criteria and then allocated to the Hugo (*n* = 34) and Spleen 6 (*n* = 34) acupressure groups using a randomized block design (six blocks). The data collection process took place from November 2022 to April 2023. The participants were blinded; however, the analysts and investigators were not blinded. Acupressure interventions were applied bilaterally for 20 min, consisting of 10 s of pressure followed by 2 s of rest. Pain intensity was assessed using a visual pain scale before, immediately after, and 1 h after the intervention. In total, 68 participants fulfilled the study. Data were analyzed using Statistical Package for the Social Sciences version 25 with chi‐square, Mann–Whitney, and Friedman tests.

**Results:**

Both groups exhibited a statistically significant reduction in postpartum pain intensity across all periods (*p* < 0.001). Although there was a significant difference in pain intensity between the groups before the intervention (*p* = 0.039), this distinction was not observed immediately and 1 h after the intervention (*p* ≥ 0.05). Both Hugo and Spleen's 6 acupressure interventions reduced postpartum pain intensity. No significant adverse events or side effects were observed.

**Conclusion:**

Acupressure on Spleen 6 and Hugo points helped decrease the severity of postpartum pain in primiparous women who had vaginal deliveries. Healthcare providers are encouraged to consider acupressure for postpartum pain management.

## 1 INTRODUCTION

Postpartum pain following vaginal delivery is a prevalent issue impacting millions of young women.^1^, ^2^ During the third stage of labor, when the placenta and membranes are expelled, the uterus undergoes contractions to constrict large uterine arteries, preventing postpartum hemorrhage.^3^ These contractions lead to the release of chemical mediators, including bradykinin, leukotrienes, prostaglandins, serotonin, and lactic acid, contributing to the experience of pain.^4^ Notably, the release of prostaglandins, responsible for inducing uterine contractions, emerges as the primary culprit behind postpartum pain.^5^ The intensity of postpartum pain spans from menstrual‐like cramps to severe discomfort, occasionally surpassing the pain experienced during childbirth itself.^5^ Typically, this discomfort persists for 3–4 days, although it can occasionally extend up to a week postdelivery.^3^ It was reported that 47% of women experienced postpartum uterus pain within 6–48 h after delivery.^6^ Pourmaleky et al. stated that 77% of women experience postpartum pain.^7^ Pain stands out as one of the most prevalent and distressing sensory and psychological ordeals.^8^ The repercussions of pain extend beyond the physical realm, impacting a mother's ability to breastfeed, engage in daily activities, and even communicate effectively with healthcare providers and her newborn.^9^ Furthermore, pain‐induced stress triggers an increase in adrenaline secretion, leading to decreased oxytocin production, which can disrupt the flow of breast milk.^10^ Therefore, pain management in the postpartum period is fundamental.^11^ Recent research indicates a range of methods for alleviating postpartum pain, encompassing massage therapy, reflexology, heat therapy, relaxation techniques, skin stimulation, herbal remedies, and pharmaceutical interventions.^12^, ^13^ Among these, oral pain relievers like mefenamic acid, ibuprofen, and acetaminophen stand out as the most commonly employed means to mitigate postpartum pain.^13^ Despite their notable efficacy in pain reduction, it is crucial to acknowledge potential side effects associated with these medications, including but not limited to nausea, vomiting, diarrhea, abdominal pain, gastrointestinal bleeding, dizziness, and drowsiness.^4^

Acupressure, a derivative of acupuncture, involves stimulating acupuncture points through finger pressure and massage to regulate and expedite bodily functions.^14^ Acupressure operates by activating specific acupuncture points to modulate pain gate control. By stimulating large nerve fibers that transmit impulses to the spinal cord, acupressure effectively closes the gates of pain transmission, thereby diminishing the perception of pain.^15^ Additionally, according to traditional Chinese medicine, the body's vital energy, or Qi, flows through meridians, governing bodily functions. Disruptions in this energy flow lead to discomfort and pain. Targeting specific points in the body allows access to these meridians, restoring balance and alleviating pain.^16^ Certain pressure points are also believed to stimulate oxytocin release and expedite labor while concurrently fostering energy equilibrium and pain reduction.^17^

A key acupressure point is Spleen 6 (SP6), positioned four fingers above the inner ankle behind the tibia's posterior edge.^18^ Wu et al. have conducted a study on the effects of acupuncture (SP6) on postcesarean section pain and reported that the acupuncture group's pain scores were lower than the control group's, and there were significant differences in the visual analog scale (VAS) scores between the acupuncture group and the control group within the first 2 h after cesarean section.^19^

The Hugo point, or Large Intestine 4 (LI4), located between the thumb and index finger, is another pivotal pressure point and one of the most common points.^13^ Afravi et al. reported that Hugo point pressure is a simple and cost‐effective, harmless, and easily applicable analgesic method for after‐pain reduction, especially in the first 2 h after delivery.^13^ Negahban Bonabi et al.'s study demonstrated a significant reduction in postcesarean section pain through acupressure at Hugo's point. The observed difference in pain intensity between the intervention and control groups was particularly notable 1 h after the intervention.^20^ Acupressure stands as a widely utilized method for alleviating postpartum pain.^21^ Despite extensive research on pain relief during childbirth, postpartum pain has been relatively underexplored.^22^

A systematic review study showed that there is currently no standard for acupressure point location, frequency, and duration of use of this method to reduce pain. Also, the durability of the relief effect of this intervention is not apparent. In this regard, the researchers have recommended more studies to investigate the preferred point of acupressure for postpartum pain and to determine the duration of the intervention's effect.^23^ The results of a systematic review showed that Hugo and Spleen's pressure points are the most used.^24^ In a study, the effect of the Hugo acupressure point and the six spleen on the severity of labor pain in primiparous women was compared, and no significant difference was observed between these two points in reducing labor pain.^25^ In another study that compared the effect of acupressure on Hugo point and six spleens on postcesarean section pain, the results showed that Hugo point acupressure compared to six spleen point had a better performance in reducing postcesarean pain.^20^

Aligned with the World Health Organization's stance on mother friendly hospitals, alleviating postpartum pain is a fundamental principle. Nonpharmacological methods, devoid of adverse effects on both mother and fetus, are preferred by patients. In Iran, Spleen 6 and Hugo are common pressure points employed for this purpose, yet there is no consensus on the preferred point of pressure. In addition, comparative studies on their efficacy in postpartum pain relief after natural childbirth are lacking.

Given the significance of postpartum pain and its direct impact on maternal satisfaction, recognizing the widespread acceptance of nonpharmacological pain reduction methods, and the significance of finding the best point for acupressure, this study was conducted to compare the effects of acupressure on the Sixth Spleen point and Hugo point on postpartum pain in primiparous women who gave birth in Malekan city in 2022.

## 2 METHODS

### 2.1 Study design

This single‐center, single‐blind, parallel randomized (1:1) trial study was conducted in Malekan, Iran, from November 2022 to April 2023.

### 2.2 Participants

**Figure 1:**
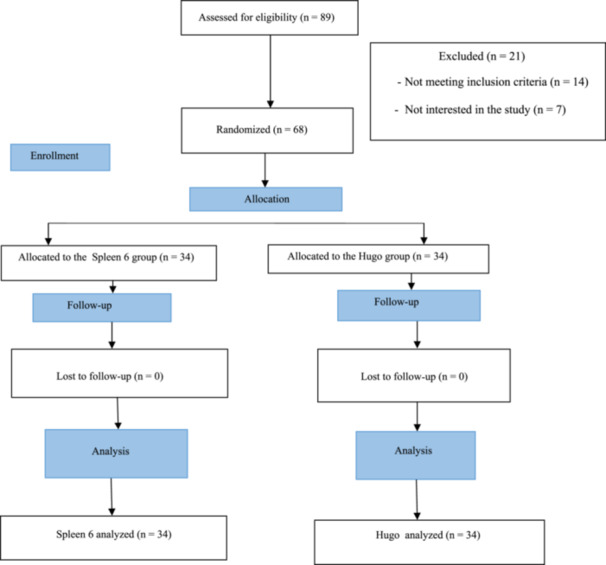
Consort flowchart of the study.

In this study, 68 eligible primiparous women who experienced postpartum pain referred to Farabi Hospital in Malekan (a city in East Azarbaijan Province in Iran) were selected according to inclusion/exclusion criteria and then randomized into two groups using a randomized block design (six blocks) (Figure 1).

### 2.3 Sample size calculation

The sample size was calculated for each group based on the results of a previous similar study.^20^ The following equation was used to calculate the sample size:

$$\frac{{\left(Z_{1-\frac{\alpha }{2}}+Z_{1-\beta }\right)}^{2}(\delta_{1}^{2}+\delta_{2}^{2})}{{\left(\mu_{1}-\mu_{2}\right)}^{2}}$$

In this equation, α = 0.05, β = 0.2, δ1 = 2.39, δ2 = 1.93, µ1 = 5.91, µ2 = 7.39, and *n* = 34. Finally, 68 participants were included in the study, with 34 participants in each group.

### 2.4 Randomization

Patients from the postpartum ward at Farabi Hospital in Malekan, located in the East Azarbaijan Province of Iran, were selected based on specific inclusion and exclusion criteria before being randomly allocated into the Hugo or SP‐6 groups at a 1:1 ratio. The allocation of participants to the Hugo or SP‐6 group was achieved through block randomization, comprising six blocks. A third party, not involved in patient diagnosis or evaluation, generated a randomized sequence via the website https://www.sealedenvelope.com. This procedure entailed the creation of 12 blocks of six, adhering to the predetermined sample size. Each participant was assigned a unique code generated through the website above, ensuring allocation concealment. Consequently, individuals involved in the study remained unaware of the next person's group assignment and were oblivious to the random sequence. This methodology upheld the study's integrity by mitigating biases related to group allocation. In this study, the participants were blinded; however, the analysts and investigators were not. A midwife from the postpartum ward was tasked with screening eligible patients according to the admission criteria and assigning random numbers. The first author (Saba Mohamadghasem‐Nejad Maleki), under the guidance of the third author (Rojin Nikrai), executed the Hugo and SP6 interventions for the respective groups. Additionally, blinded to the group allocations, another midwife received training to collect data.

### 2.5 Inclusion and exclusion criteria

The inclusion criteria comprised being a primiparous woman (first‐time mother), having undergone an episiotomy, and possessing no lesions at the intended site of acupressure application. The exclusion criteria were defined as follows: a lack of willingness to participate; prior experience with acupressure; any speech, hearing, or visual impairments; a history of substance abuse; diagnosed mental health disorders; and the occurrence of complications such as significant hemorrhage, embolism, or the necessity for analgesics within the initial 2 h postpartum.

### 2.6 Data collection

The demographic questionnaire was administered through face‐to‐face interviews, while pain intensity was assessed using the VAS at three time points: before the intervention, immediately afterward (within 5 min), and 1‐h postintervention. This assessment was conducted by a third party not involved in the study. The VAS employs a 10 cm graduated line, where a score of 10 indicates the most severe pain and 0 denotes the absence of pain. The patient's mark along this line determines their pain level, with pain severity categorized as mild (1–3), moderate (4–7), or severe (8–10).^20^ The VAS is recognized for its validity and scientific reliability.^22^

The content validity method was employed to ensure the demographic questionnaire's validity. This process entailed aligning the questionnaire with the research objectives incorporating insights from scientific literature, articles, and studies by other researchers. The supervising and consulting faculty reviewed, approved, and modified the draft form. It was subsequently presented for final review and approval to 10 academic staff members from the Faculty of Nursing and Midwifery at Kermanshah University of Medical Sciences, including 2 female obstetrics specialists, 1 midwife, and 7 professors with expertise in midwifery and reproductive health. Following comprehensive discussions and revisions by this panel of experts, the refined questionnaire was adopted for the study. This meticulous validation process, incorporating diverse academic insights, significantly enhances the questionnaire's content validity, ensuring its congruence with the research goals and adherence to scientific standards.

### 2.7 Intervention

**Figure 2:**
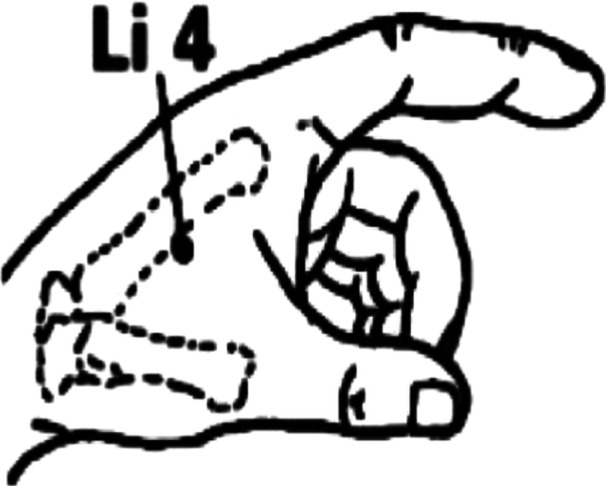
Hugo point. Li4, large intestine 4.

**Figure 3:**
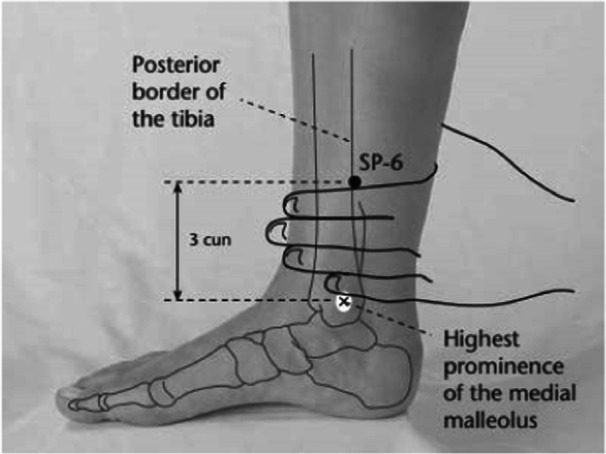
Spleen 6 (SP‐6) point.

Interventions were delivered to primiparous mothers immediately following natural childbirth, both directly after delivery and upon their transfer to the postpartum ward. These procedures were conducted before the initiation of breastfeeding, within a 2‐h window postdelivery. Acupressure was applied bilaterally at Spleen 6 point (Figure 2) and Hugo point (Figure 3). For each point, the pressure was applied for 10 s, followed by a 2‐s rest, over a continuous duration of 20 min. Under the guidance of the third author, the first author administered the interventions, following a protocol adapted from the Negahban Bonabi study.^20^

The pressure intensity was adjusted to produce a sensation of warmth and mild discomfort. The Spleen 6 point is located 5 cm above the inner ankle along the Spleen meridian, whereas the Hugo point is found on the back of the hand, between the first and second metacarpal bones, in alignment with the radial bone. In addition to the acupressure intervention, all participants received standard postpartum care, which included monitoring of vital signs, uterine massage, and bleeding control measures. Furthermore, in the postpartum ward, women who requested it were administered a 100 mg Diclofenac suppository at 4 and 10 h postdelivery.

### 2.8 Outcome measures

#### 2.8.1 Primary outcome

The primary outcome was the severity of postpartum pain.

### 2.9 Statistical analysis

The statistical analysis utilized the Statistical Package for the Social Sciences, version 25.0. The Kolmogorov–Smirnov (KS) test determined the normality of data distribution. The chi‐square test assessed the differences in the frequency of qualitative variables between the two groups. To examine the trends in mean scores of pain intensity (total score) before, immediately after, and 1 h after the intervention across the groups, Friedman's test (the nonparametric equivalent of the repeated measures test) was employed. The Mann–Whitney *U* test (the nonparametric counterpart to the independent *t*‐test) compared the mean pain intensity scores (total score) at different time intervals between the Hugo and SP‐6 groups. The intention‐to‐treat analysis model was applied to data analysis. A significance level, or *p*‐value, of less than 0.05 was set for all analyses.

### 2.10 Ethical considerations

Initially, all participants were informed about the study's details and provided their consent by signing an informed consent form. The study received approval from the Ethics Committee of Kermanshah University of Medical Sciences and was registered in the Iranian Registry of Clinical Trials under the code [IRCT20220910055926N1].

## 3 RESULTS

In total, 68 primiparous women who met the inclusion criteria were randomly divided into two groups (*n* = 34 each): the Spleen 6 and Hugo groups. The Kolmogorov–Smirnov test assessed the normality of quantitative variables within both groups. A comparative analysis of demographic variables between the Hugo and Spleen 6 groups showed no statistically significant differences in demographic factors. The Mann–Whitney test, applied to evaluate the average age difference given the nonnormal distribution in the Spleen 6 group, indicated no significant age difference between groups (*p* = 0.212) (Table 1).

**Table 1:** Demographic characteristics of participants in the Hugo and six spleen groups.

Variables		Hugo intervention group	Six spleen intervention group	*p*‐value
Maternal education	Under diploma	20 (58.8)	19 (55.9)	0.806^a^
	Diploma and upper	14 (41.2)	15 (44.1)	
Job	Housewife	25 (73.5)	28 (82.4)	0.380^a^
	Employee and others	9 (26.5)	6 (17.6)	
Residence	Personal	8 (23.5)	8 (23.5)	0.698^a^
	Rental	9 (26.5)	12 (35.3)	
	Family	17 (50)	14 (41.2)	
Childbirth class attendance	Yes	10 (29.4)	6 (17.6)	0.253^a^
	No	24 (70.6)	28 (82.4)	
Breastfeeding	Yes	29 (85.3)	27 (79.4)	0.525^a^
	No	5 (14.7)	7 (20.6)	
Baby gender	Boy	17 (50)	15 (44.1)	0.627^a^
	Girl	17 (50)	19 (55.9)	
Age (Mean ± SD)		28.82 ± 6.39	27.18 ± 6.96	0.212^b^

a Chi‐square tests.

b Mann–Whitney *U*.

The Kolmogorov–Smirnov test demonstrated that pain intensity variables were not normally distributed before, immediately after, and 1 h after the intervention in both groups. The nonparametric Mann–Whitney test compared pain intensity scores across the three time points between the groups, revealing a statistically significant difference in pain intensity scores before the intervention. However, no significant differences were observed immediately after and 1 h after the intervention (Table 2).

**Table 2:** Comparison of pain intensity at three time points in the study groups.

Times	Hugo point group mean ± SD	Six spleen point group mean ± SD	*p*‐value
Before	7.79 ± 1.20	7.18 ± 1.23	0.039^a^
Immediately after the intervention	5.79 ± 1.69	6.33 ± 1.26	0.400^a^
1 h after the intervention	4.56 ± 1.60	4.61 ± 1.44	0.690^a^
*p*‐value	*p* < 0.001^b^	*p* < 0.001^b^	

a Mann–Whitney *U* test

b Friedman test.

Due to the nonnormal distribution of pain intensity variables, the Friedman test was employed to examine the trends in pain intensity across three time intervals. This analysis showed a statistically significant reduction in pain intensity over time in both the Hugo and Spleen 6 acupressure groups (*p* < 0.001) (Table 2). No significant adverse events or side effects were reported.

## 4 DISCUSSION

The findings of this study demonstrate a statistically significant reduction in postpartum pain intensity across three time intervals (before, immediately after, and 1 h after the intervention) in the six spleen acupressure point group. Consistent with our results, Wu et al. have conducted a study on the effects of acupuncture on postcesarean section pain and reported that the acupuncture group's pain scores were lower than the control group's, and there were significant differences in the VAS scores between the acupuncture group and the control group within the first 2 h after cesarean section.^19^

Lee et al.^26^ conducted a study examining the impact of acupressure on the acupuncture point SP‐6. In their research involving 75 women, 36 received acupressure at the SP‐6 point, while 39 received only tactile touch at the same point. Acupressure was administered for 30 min, and pain intensity was assessed 30 and 60 min after the intervention. Similar to the findings in the present study, Lee et al. reported that women who underwent acupressure exhibited reduced pain levels.^26^

Halime and Okumus reported lower pain levels in the intervention group compared to the control group following acupressure at six spleen points.^27^ Negahban Bonabi et al. did not observe a statistically significant difference in mean postpain scores after cesarean section between the six spleen acupressure and control groups.^20^ Similarly, Soltani et al. found no significant differences in uterine tonicity and pain 1 and 2 h after delivery among groups receiving acupressure on main points, sham acupressure, and control.^28^ Additionally, Adib‐Hajbaghery et al. reported no significant reduction in pain levels, nausea, and vomiting after appendectomy with acupressure at the spleen point.^29^ The precise mechanism of pain reduction via acupressure and acupuncture at the SP‐6 point remains unclear. It is theorized that acupuncture modulates the nervous system, influencing input signals to the central nervous system. This activation may engage pain‐regulating systems, including internal opioid pathways. Studies have shown elevated endorphin levels in cerebrospinal fluid and the brain after acupuncture, implying their potential role in pain alleviation.^30^

The results of this study suggest that acupressure at the Hugo point significantly reduced the average intensity of postpartum pain across three critical time intervals: before, immediately after, and 1 h after the intervention. These findings align with the study by Negahban Bonabi et al., which suggests a statistically significant difference in after‐pain scores following cesarean section in the Hugo point acupressure group compared to the control group, showcasing the efficacy of Hugo point acupressure in alleviating postoperative pain.^20^ Hamidzadeh et al.'s study also provided consistent results, illustrating that acupressure at the Hugo point effectively reduced labor pain compared to the control group.^31^ In a study by Kumar and Viswanath,^32^ significant differences in pain scores were observed between groups at 30 and 60 min postintervention. Women in the acupressure group reported a positive experience, suggesting that Hugo Point acupressure is a cost‐effective nursing intervention for enhancing comfort during childbirth.^32^ Smith et al.'s study further supports these findings, demonstrating that acupressure effectively reduces labor pain.^33^ The study by Ganji et al. emphasized the reliability of the six spleens and Hugo points for labor pain reduction in their review, as they were consistently employed in studies with acceptable validity.^24^

However, it is worth noting that some studies present contradictory results. For instance, the study by Ramezani et al. in 2016 showed that acupressure at Hugo's point had no discernible effect on reducing postoperative pain after cesarean surgery.^34^ In a study by Yeh et al. comparing the effects of auricular point acupressure with painkillers on postspine surgery pain, ear acupressure did not prove effective in pain reduction.^35^ These outcomes may be attributed to variations in acupressure or acupuncture techniques, including the application of electrical acupressure. Additionally, the selection of control groups and the specific context of the surgical procedure can play a role in influencing the observed effects. Moreover, postoperative pain is a multifaceted phenomenon influenced by various factors, such as age, personality traits, education, social status, and the patient's awareness and understanding of the surgical process, medical care, time of day, and physical condition.^19^

The results of this study indicate no significant difference in the average intensity of postpartum pain between the two acupressure groups (six spleen points and Hugo) immediately after and 1 h after the intervention.

In some studies, age has been reported as a factor affecting pain intensity.^36^, ^37^ However, in our study, a thorough examination of the studied groups regarding age and education before the test indicated homogeneity, allowing us to attribute changes in average pain intensity to the interventions. The exact mechanism underlying acupressure's effect on pain remains unknown. It is suggested that acupressure, by stimulating energy channels, establishes a balance between forces and energy flow. It may also hinder the transmission of pain signals and elevate endorphin levels.^26^ Furthermore, reducing anxiety levels may contribute to pain reduction, as anxiety is associated with increased catecholamines, leading to decreased endorphins, heightened pain, and prolonged labor.^38^ Another possible mechanism is the “gate control theory of pain,” which posits that pressure stimulates large nerve fibers, ultimately keeping pain transmission gates closed and reducing pain perception.^39^ According to Chinese medicine, energy channels known as meridians exist within the body, and blockages in these channels lead to imbalances in energy, potentially resulting in pain during childbirth. Therefore, stimulating these points may restore energy balance and alleviate pain.^40^

## 5 LIMITATIONS OF THE STUDY

This study has several limitations that may affect the generalizability of its findings. Pain is subjective, with psychological factors significantly influencing its perception and manifestation. Factors such as underlying mood disorders, emotional support, fatigue, and past trauma can affect pain outcomes,^41^, ^42^, ^43^, ^44^ introducing variability that the study may not fully account for. The reliance on the visual pain scale and self‐reported data for pain assessment, without objective criteria, introduces potential bias due to individual interpretation variations. The study's exclusive enrollment of women with standard deliveries limits the findings' applicability to a broader population experiencing various delivery methods.

Additionally, focusing on postpartum pain within the hospital setting without long‐term follow‐up restricts understanding of pain's persistence or evolution postdischarge. The absence of a control group without acupressure treatment limits the study's ability to make direct comparisons and attribute observed changes to the acupressure intervention. Furthermore, the impact of acupressure on the need for painkillers postintervention was not investigated, representing another research limitation.

## 6 CONCLUSION AND RECOMMENDATIONS

The study indicates a statistically significant reduction in postpartum pain intensity across three time intervals (before, immediately after, and 1 h after the intervention) for both the Hugo and Spleen 6 acupressure groups. These findings suggest that acupressure at these points can effectively reduce postpartum pain intensity in women following natural childbirth. Healthcare providers, including midwives and gynecologists, are encouraged to consider acupressure on the Hugo and Spleen 6 points as part of their care protocol for managing postpartum pain.

## AUTHOR CONTRIBUTIONS

**Saba Mohamadghasem‐Nejad Maleki**: Writing—original draft; methodology; investigation. **Sousan Heydarpour**: Conceptualization; methodology; writing—review and editing; supervision; project administration; visualization. **Rojin Nikrai**: Methodology; writing—review and editing. **Fateme Heydarpour**: Methodology; formal analysis; validation.

## CONFLICT OF INTEREST STATEMENT

The authors declare no conflict of interest.

## ETHICS STATEMENT

The Ethics Committee of Kermanshah University of Medical Sciences approved the study with code [IR.KUMS.REC.1401.251].

## TRANSPARENCY STATEMENT

The lead author Sousan Heydarpour affirms that this manuscript is an honest, accurate, and transparent account of the study being reported; that no important aspects of the study have been omitted; and that any discrepancies from the study as planned (and, if relevant, registered) have been explained.

## Data Availability

The corresponding author had full access to all study data and was responsible for data integrity and analysis accuracy.
